# Prevalence and risk factors of obstructive sleep apnoea in people with sarcoidosis: a narrative review

**DOI:** 10.1007/s11325-025-03493-y

**Published:** 2025-11-01

**Authors:** Gianluca Cotta, Steven Luu, Brendon J. Yee, Edmund M.T. Lau, Diego Garcia-Borreguero

**Affiliations:** 1 Sleep & Circadian Research Group, Woolcock Institute of Medical Research, Macquarie University, 2 Innovation Rd, Macquarie Park, NSW 2113 Australia; 2 Department of Respiratory and Sleep Medicine, Royal Prince Alfred Hospital, Camperdown, NSW Australia; 3 Central Clinical School, Sydney Medical School, University of Sydney, Sydney, Australia; 4 Sleep Research Institute, 28036 Madrid, Spain

**Keywords:** Sarcoidosis, Obstructive sleep apnoea, Corticosteroid therapy

## Abstract

**Background:**

Emerging evidence suggests a link between sarcoidosis and obstructive sleep apnoea (OSA). Previous studies have reported a high prevalence of OSA in people with sarcoidosis; however, much of this evidence has been derived from retrospective analyses. Recently, prospective cohort studies have provided further insight into potential interactions between sarcoidosis and OSA. This narrative review aims to synthesise current evidence on the prevalence of OSA in people with sarcoidosis and identify associated risk factors.

**Methods:**

We conducted a search of Pubmed, Medline, the Cochrane Library and the Register of Controlled Trials and identified eight studies that evaluated the prevalence of OSA in people with sarcoidosis.

**Results:**

The prevalence of OSA in sarcoidosis patients ranged from 44% to 88%. Mild OSA was generally more common than moderate to severe OSA, with prevalence rates of 24% to 52% for mild cases and 5% to 51% for moderate to severe cases. Advancing age and elevated body mass index (BMI) were consistently associated with increased OSA risk. Corticosteroid therapy and sex showed mixed associations, while parenchymal lung involvement was not linked to increased OSA risk in any study.

**Conclusion:**

The prevalence of OSA in people with sarcoidosis is high. Conventional risk factors for OSA, including age and BMI, also apply in this group. Further prospective studies with larger, well-defined cohorts are warranted to clarify these associations and explore the potential bidirectional relationship between sarcoidosis and OSA. Studies should expand our knowledge on symptoms and hypoxic burden of subjects with sarcoidosis and OSA.

## Introduction

Sarcoidosis is a multisystem inflammatory disease characterised by the formation of non-caseating epithelioid granulomas, which distort the normal micro-architecture of affected tissues [1]. The prevalence of sarcoidosis is regionally heterogeneous, ranging from as low as 1–5 per 100,000 in some countries to as high as 140–160 per 100,000 in others [2]. While sarcoidosis can affect any organ, parenchymal lung involvement is most common. Obstructive sleep apnoea (OSA), characterised by repetitive episodes of upper airway collapse during sleep, has been reported at higher rates in people with sarcoidosis than in the general population based on small observational studies [3]. Sarcoidosis and OSA share overlapping symptoms and clinical features, including fatigue, hypersomnolence, cognitive deficits and pulmonary hypertension. Notably, people with comorbid pulmonary sarcoidosis and OSA have a higher apnoea-hypopnoea index (AHI) compared to those with comorbid non-pulmonary sarcoidosis and OSA [4]. Both disorders are characterised by a proinflammatory state, interactions of which are still unknown in terms of long-lasting cardiovascular or neurological outcomes.

Emerging evidence suggests a potential link between sarcoidosis and an increased risk of OSA, and multiple mechanisms have been hypothesised. Sarcoid lesions in the upper respiratory tract (SURT) may directly increase upper airway resistance, leading to OSA [5]. Likewise, pulmonary sarcoidosis can reduce lung volumes and further elevate upper airway resistance [6]. Sarcoidosis-associated myopathy or neuropathy may lead to increased upper airway hypotonia and collapsibility.[3] Corticosteroid therapy, commonly used in sarcoidosis management, may exacerbate these mechanisms by inducing weight gain and increasing fat deposition around the upper airway, resulting in increased upper airway collapsibility [7].

The link between sarcoidosis and OSA has been examined sporadically over the past three decades. A case-control study in 1997 assessed the prevalence of OSA in people with sarcoidosis compared to patients from a general pulmonary clinic [8]. While that study supported a higher prevalence of OSA among people with sarcoidosis, its findings were limited by a small and heterogeneous sample. A more recent study observed a strong association between sarcoidosis and excessive daytime sleepiness in a large cohort of patients (*N*=1067), but it did not specifically evaluate the prevalence or severity of OSA in this population [9]. In 2015, a review aimed to synthesise the available evidence of sleep-disordered breathing in sarcoidosis [3]. Although it concluded there was a higher prevalence of OSA in people with sarcoidosis, it highlighted the methodological limitations of existing studies – most being observational, involving small sample sizes, or both.

Since then, additional observational studies have been conducted, offering further insights into the interactions between sarcoidosis and OSA. Our review aims to synthesise the current body of evidence to determine the prevalence of OSA in people with sarcoidosis and to identify potential risk factors that may contribute to its development.

## Methods

### Search strategy

A literature search was conducted in Pubmed, Medline, the Cochrane Library and the Register of Controlled Trials to identify relevant studies published from database inception through December 2024. We used MeSH and free-text terms including ‘sarcoidosis’, ‘obstructive sleep apnoea’, ‘sleep-disordered breathing’, ‘prevalence’, and ‘risk factors’. Reference lists of key articles and reviews were manually screened to identify additional eligible studies. Additional searches were conducted using Google Scholar and grey literature sources to ensure the inclusion of potentially relevant studies missed by our initial search. The search terms were developed collaboratively by the authors; however, the literature screening and study selection were performed by a single author (G.C.).

### Eligibility criteria

Studies were included if they met the following criteria: (1) participants had a confirmed diagnosis of sarcoidosis clinically or based on the American Thoracic Society (ATS)/European Respiratory society (ERS) guidelines 2) OSA prevalence was assessed and diagnosis was established by either polysomnography (PSG) or a validated home sleep apnoea test (HSAT); and (3) specified whether corticosteroid therapy was prescribed as part of sarcoidosis management. Studies were excluded if data were collected retrospectively, due to potential misclassification bias [10], or if they had fewer than 15 participants with sarcoidosis.

## Results

### Characteristics of eligible studies

A total of eight studies met the inclusion criteria and their key characteristics are summarised in Table 1 [11–18]. Participants across the studies were generally overweight or obese (body mass index (BMI) range 25.7 to 31.0 kg/m^2^). Mean age of participants varied across studies ranging from 43.9 to 60.3 years old. Most participants had sarcoidosis classified as Scadding radiographic stages I to III [19] however extrapulmonary sarcoidosis was inconsistently reported. Cardiac sarcoidosis was reported in one study, where it was present in 30% of participants [12]. Treatment with corticosteroids was prescribed to less than 50% of participants in several studies [11, 13, 16, 18]. Two studies diagnosed OSA using HSAT [12, 13] while the remainder of the studies used in-lab PSG [11, 14, 18]. All studies enrolled participants from either a tertiary or university hospital setting, with two studies enrolling participants consecutively diagnosed with sarcoidosis [12, 17, 18]. Most studies lacked a healthy control group and did not report participation rates. Three studies did not stratify the results according to sex, BMI, and age due to limited sample sizes [12, 15, 17]. Polysomnographic data of the cohorts are summarised in Table 2. The average Epworth sleepiness scale (ESS) score among people with sarcoidosis was below 10 in all studies, suggesting that excessive daytime sleepiness was not a predominant clinical feature [20].

**Table 1:** Characteristics of eligible studies

Study	Location	Type of study	Sample size	Study population	Mean age (years)	Male/Female	Mean BMI (kg/m2)	Sarcoidosis diagnostic criteria	Scadding stage of sarcoidosis	Extrapulmonary sarcoidosis	Time of recollection	Exclusion criteria	Healthy subject included	Participation rate
Ataoğlu, 2022 [11]	Turkey	Cross-sectional	54	Tertiary respiratory clinic (consecutive recruitment)	52	11/43	31.0	Clinical	Stage I: 15% Stage II: 67% Stage III: 8%	NR	01/2019-12/2019	1–2-3	NA	NR
Roeder, 2022 [12]	Switzerland	Cross-sectional	71	Tertiary hospital visits and sarcoidosis database	50	38/33	25.7	ATS/ERS criteria	Stage I: 23% Stage II: 72% Stage III-IV: 5%	69%	10/2019-10/2021	2–4-5–6-7	1-to-1 match	77%
Mari, 2020 [13]	Italy	Cross-sectional	68	Tertiary sarcoidosis clinic	60.3	26/42	27.9	ATS/ERS criteria	Stage 0-I: 41% Stage II-IV: 59%	44%	04/2019-07/2019	2–5	NA	81%
Doğan, 2019 [14]	Turkey	Cross-sectional	46	University respiratory clinic	44.4	11/35	29.3	Histopathology	Stage I: 59% Stage II: 41%	28.2%	01/2019-12/2019	1–2-3–8	NA	NR
Mavroudi, 2017 [15]	Greece	Cross-sectional	21	Tertiary hospital respiratory clinic	53.8	7/14	28.6	Clinical and radiological	Stage I-II: 100%	NR	11/2013-11/2015	1–2-3–4-9–10	15	85%
Bingol, 2015 [16]	Turkey	Cross-sectional	29	University ILD clinic (consecutive recruitment)	43.9	2/27	26.8	Histopathology	Stage I: 48% Stage II-III: 52%	NR	11/2009-02/2010	1–2-3–10-11–12-13	NA	NR
Pihtili, 2013 [17]	Turkey	Cross-sectional	15	University respiratory clinic (consecutive recruitment)	46.4	1/14	26.4	Histopathology	Stage II-III: 100%	NR	11/2009-02/2010	1–2-3–10-11–12-13	NA	NR
Verbraecken, 2004 [18]	Belgium	Cross-sectional	46	Tertiary hospital clinic	45	26/20	29	International guidelines	Stage 0: 17% Stage I: 11% Stage II: 26% Stage III-IV: 26%	NR	01–1999-08/2003	2–4-11	NA	NR

1 = Current intake of medication that interferes with sleep architecture (antihistamines, antidepressants, hypnotics); 2 = confirmed diagnoses of psychiatric and neurological conditions; 3 = previous upper respiratory tract infection in the previous three months; 4 = morbid or severe disease prohibiting protocol adherence; 5 = CPAP treatment for OSA at baseline; 6 = use of supplemental oxygen; 7 = pregnancy; 8 = current steroid therapy; 9 = alcohol abuse; 10 = total sleep less than 4 hours; 11 = other chronic respiratory conditions; 12 = BMI>30; 13 = Mallampati score 3–4; *BMI* body mass index, *ILD* interstitial lung disease, *NR* not reported, *NA* not applicable

**Table 2:** Sleep characteristics of people with sarcoidosis

Study	Sleep study type	ESS score	AHI (events/h)	ODI (events/h)	Mean SpO_2_ (%)	T90 (%)
Ataoğlu,2022 [11]	PSG	NR	13.1 (13.1)	14.6 (15.3)	NR	9.6 (17.4)
Roeder,2022 [12]	HAST	9 (6–11)	4 (1.3–9.3)	6.3 (2.3- 13.8)	93 (91–94)	5 (0–29)
Mari, 2020 [13]	HAST	7.9 (5.2)	NR	NR	NR	NR
Doğan, 2019 [14]	PSG	2.6 (3.2)	10.7 (13.8)	11.9 (11.8)	94.5 (2.1)	4.8 (12.6)
Mavroudi, 2017 [15]	PSG	4.3 (2.4)	5.6 (3.6)	5.2 (5.4)	93.7 (1.9)	NR
Bingol, 2015 [16]	PSG	4 (2.9)	9.6 (15.1)	13.5 (20.8)	94.4 (3.1)	NR
Pihtili, 2013 [17]	PSG	3.86 (2.6)	14.5 (19.9)	19.69 (27)	93.7 (3.6)	NR
Verbraecken, 2004 [18]	PSG	NR	3.7 (8.8)	NR	92.4 (2.5)	13.9 (20.9)

Figures are provided as mean (SD) or median (25–75 quartile). *AHI* apnoea-hyponoea index, *ESS *Epworth Sleepiness Scale, *HAST* home apnoea sleep test, *NR* not reported, *ODI* oxygen desaturation index, *PSG* polysomnography, *T90* percentage of time spent with a SpO_2_ below 90% during the night

### Prevalence of OSA in people with sarcoidosis

The reported prevalence of OSA among people with sarcoidosis ranged from 44% to 88% (Table 3) with the highest prevalence observed in the oldest cohort [13]. Mild OSA was generally more common than moderate to severe OSA in most studies, with prevalence rates ranging from 24% to 52% for mild cases and 5% to 51% for moderate-severe cases. The mean AHI of people with OSA and sarcoidosis ranged between 14.5 and 19.

**Table 3:** Prevalence of OSA in participants with sarcoidosis

Study	OSA definition	Prevalence of OSA (%)	Prevalence of mild OSA (%)	Prevalence moderate-severe OSA (%)	Mean AHI of people with OSA and sarcoidosis (events/h)
Ataoğlu,2022 [11]	ICSD-3	70	39	31	17.7 (13.1)
Roeder,2022 [12]	AHI≥5	61	45	16	NR
Mari, 2020 [13]	ICSD-3	88	37	51	NR
Doğan, 2019 [14]	AHI≥5, and a sleep apnoea symptom	61	41	20	16.3 (15.4)
Mavroudi, 2017 [15]	AHI≥5	57	52	5	NR
Bingol, 2015 [16]	AHI≥5	52	42	10	16 (19)
Pihtili, 2013 [17]	AHI≥5	67	47	20	14.5 (19.9)
Verbraecken, 2004 [18]	AHI≥5	44	24	20	19 (16)

Figures are provided as mean (SD). *AHI* apnoea-hypopnoea index, *ICSD-3* International Classification of Sleep Disorders Third Edition, *NR* not reported, *OSA* obstructive sleep apnoea

### Comparison of clinical characteristics in people with sarcoidosis with and without OSA

People with sarcoidosis and concomitant OSA were older and had a higher BMI than people with sarcoidosis and no OSA (Table 4). One study reported a higher prevalence of upper respiratory tract obstruction in patients with sarcoidosis and OSA, though not statistically significant [11]. Findings regarding corticosteroid therapy and sex distribution were mixed.

**Table 4:** Comparison of clinical characteristics in people with sarcoidosis with and without OSA

Study	Average age (with OSA vs without OSA) (years)	Percentage of female (with OSA vs without OSA)	Average BMI (with OSA vs without OSA)	Percentage receiving corticosteroids (with OSA vs without OSA)	Percentage with an upper respiratory tract obstruction (with OSA vs without OSA)	Average predicted FEV_1_ (with OSA vs without OSA) (%)
Ataoğlu, 2022 [11]	54 (11) vs 47 (13)†	24% vs 55%^+^	31.9 (4) vs 29 (5)†	55% vs 19%†	44% vs 15%	99 (11) vs 92 (17)
Roeder, 2022 [12]	NR‡	NR (male sex OR 3.16; 95% CI 1.18–8.45)	NR (OR 1.17; 95% CI 1.02–1.33; *P* =.022)	NR‡	NR	NR‡
Mari, 2020 [13]	Mild OSA 61.5 (10.5), moderate-severe OSA 61 (10.1) vs 53.2 (5.7)	87% vs 58%†	Mild OSA 26.2 (3.1), moderate-severe OSA 29.5 (5.1) vs 26.2 (8.3) †	Mild OSA 40%, moderate-severe OSA 54% vs 25%	NR	NR
Doğan, 2019 [14]	47.8 (9.3) vs 38.9 (10.5)†	78% vs 72%	30.3 (4.4) vs 27.6 (5.6)	NA	NR	94.3 (18.70) vs 98.3 (13.8)
Mavroudi, 2017 [15]	NR	NR	NR	NR	NR	NR
Bingol, 2015 [16]	NR	NR	NR	NR‡	NR	NR
Pihtili, 2013 [17]	NR	NR	NR	NR	NR	NR
Verbraecken, 2004 [18]	50 (13) vs 40 (7)†	40% vs 46%	32 (5) vs 27 (6)†	NR	NR	85 (15) vs 78 (24)

Figures are provided as mean (SD) or median (25–75 quartile). † = Statistically significant as specified; ‡ = numeric values not reported; however not statistically significant; *BMI body mass index*, *CI* confidence interval, *FEV*_*1*_ forced expiratory volume in 1 second, *NR* not reported, *NA* not applicable, *OR *odds ratio, *OSA* obstructive sleep apnoea

Two studies demonstrated a higher prevalence of OSA in patients with sarcoidosis receiving concomitant corticosteroid therapy [11, 13]. The remaining studies either did not report differences in corticosteroid use between patients with and without OSA or found that such differences were not statistically significant. One study reported a significant female predominance among sarcoidosis patients with OSA [13], whereas another found a greater proportion of males in the OSA group [11]. Other studies did not observe any significant association between sex and OSA prevalence. Pulmonary function test results were generally within normal physiological limits across both groups, with no consistent or clinically meaningful differences.

## Discussion

While earlier retrospective analyses identified an increased prevalence of OSA among people with sarcoidosis [8, 9], our review extends these findings by demonstrating a prevalence ranging from 44% to 88% across the included studies. This significantly exceeds the reported global prevalence of OSA in the general population, estimated between 4% and 30% [21]. However, the absence of matched healthy control groups in most studies limits the ability to draw conclusions regarding comparative prevalence or causality between sarcoidosis and increased OSA risk.

The substantial variability in reported OSA prevalence likely reflects heterogeneity in study design, diagnostic methodologies, and sample demographics. Earlier studies utilised more stringent criteria for hypopnoea (≥4% oxygen desaturation and ≥50% reduction in airflow) [16–18], whilst more recent studies applied a revised definition (≥3% oxygen desaturation and ≥30% reduction in airflow) [11–14], potentially leading to higher diagnostic rates. Differences in sarcoidosis severity may also contribute. Studies with a higher proportion of patients in advanced Scadding stages may reflect greater pulmonary involvement, which could impact upper airway patency or ventilatory control. Recruitment strategies also varied, with some studies applying broad exclusion criteria and others using consecutive sampling, further influencing cohort characteristics.

Another methodological limitation concerns the classification of mild OSA. Five of the eight studies did not require that patients with an AHI between 5–15 also report clinical symptoms, as defined in the International Classification of Sleep Disorders, Third Edition, Text Revision (ICSD-3-TR) [22]. Therefore, the prevalence of mild OSA in these studies may not be directly comparable to that in other population-based studies that apply the ICSD-3-TR diagnostic criteria. In contrast, reported rates of moderate-to-severe OSA (AHI ≥15 irrespective of symptoms) ranged from 10% to 51% in sarcoidosis cohorts, compared with estimates of ~9% in women and ~17% in men aged 50–70 years in the Wisconsin Sleep Cohort [23]. These findings support an increased burden of OSA in people with sarcoidosis, although the overlapping ranges suggest the excess risk may be more modest than implied when all mild cases are included.

Differences in baseline age and BMI across cohorts may also explain discordant prevalence estimates. Among the clinical factors examined, advancing age and higher BMI were consistently associated with an elevated risk of OSA in people with sarcoidosis, aligning with well-established risk factors in the broader population [24]. Paradoxically, some epidemiological data suggest that obesity may be protective against sarcoidosis. A large cohort study from the US Veterans Health Administration reported lower sarcoidosis incidence in obese people [25]. This has been attributed to reduced interferon release, despite obesity's pro-inflammatory profile. Notably, interferon-α has been implicated in granuloma formation through pulmonary macrophage activation, a key step in sarcoidosis pathogenesis [26–28]. Other potential contributors such as corticosteroid therapy, upper respiratory tract involvement and reduced pulmonary function remain unclear due to inconsistent or limited reporting across studies.

### Possible mechanistic link between OSA and sarcoidosis

**Fig. 1:**
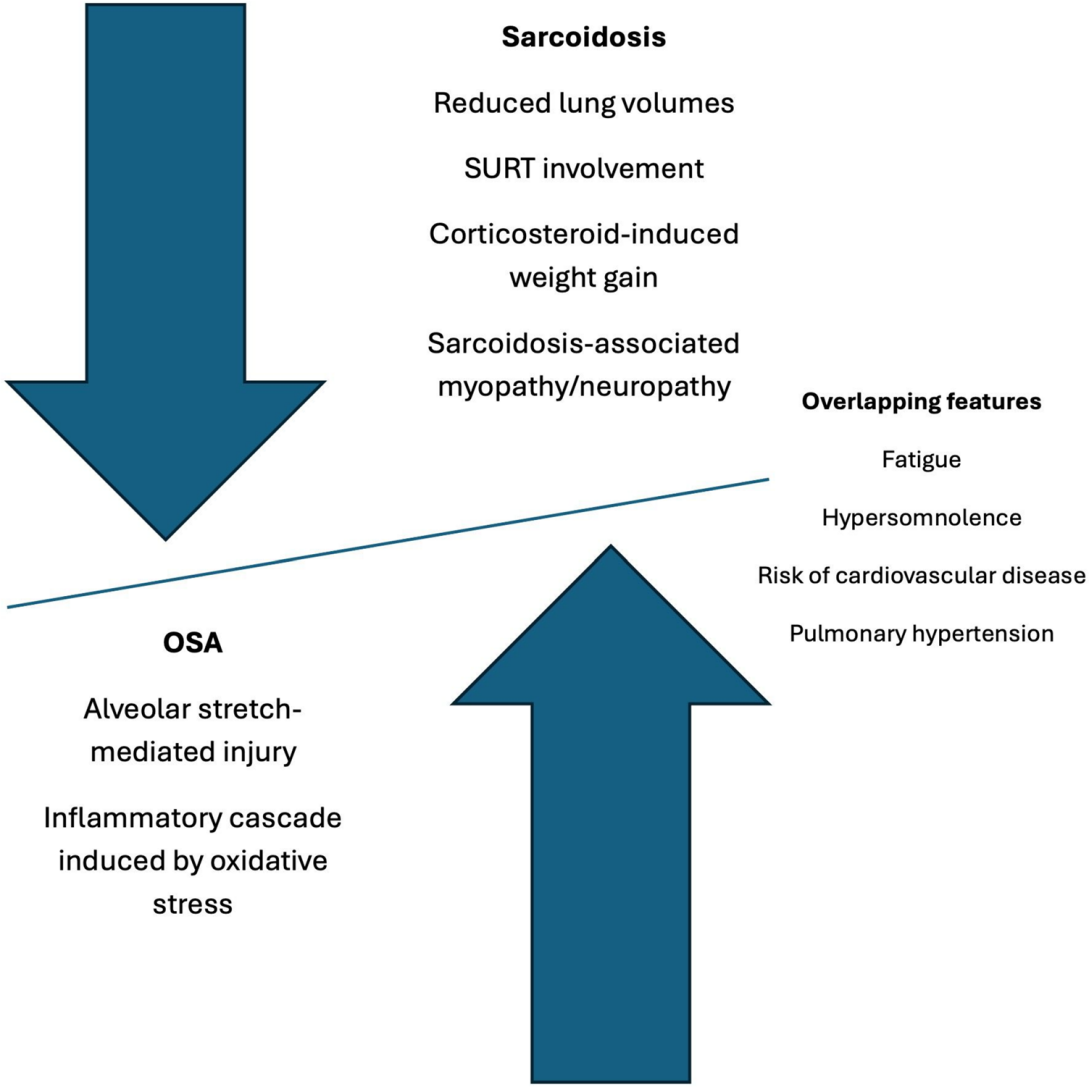
Proposed pathomechanisms linking sarcoidosis and OSA. SURT = Sarcoid lesions in the upper respiratory tract

Several physiological mechanisms have been proposed to explain the increased prevalence of OSA in people with sarcoidosis (Fig. 1), although evidence remains limited and mixed. In interstitial lung diseases (ILDs), restrictive impairment due to lung parenchymal involvement compromises upper airway stability by decreasing lung-volume induced traction [6, 29]. This is particularly pronounced during rapid eye movement (REM) sleep due to physiological atonia of intercostal muscles [30, 31]. Repetitive apnoeic events can also cause alveolar stretch-mediated injuries due to inflammation of the alveolar epithelium, potentially contributing to early interstitial lung injury [32]. In our review, two studies observed a correlation between advanced sarcoidosis (Scadding stages II-III) and elevated AHI, suggesting these mechanical factors may increase the risk of OSA in this population [11, 16].

Two studies also observed a high proportion of REM-related sleep apnoea in people with sarcoidosis and OSA, ranging between 40% to 52.9% [16, 17]. Interestingly, both cohorts featured a high proportion of female participants, consistent with the known epidemiology of sarcoidosis. While female sex is associated with lower OSA risk in the broader population, hormonal factors may alter this relationship in REM-predominant OSA. Progesterone has been shown to enhance upper airway dilator muscle activity, particularly genioglossus tone, during wakefulness. Although this can serve as a protective factor during non-REM sleep [33], its effects may be overwhelmed by muscle atonia during REM sleep [34, 35].

No significant association was observed between sarcoidosis-induced parenchymal impairment and the risk of OSA when assessing the correlation between pulmonary function measures and AHI. However, prior studies have observed a discordance between pulmonary function measures and radiological severity in almost 50% of people with sarcoidosis [36, 37]. While FVC decline may indicate chronic disease progression, it is seen in only 10–30% of cases [38]. Hence this lack of observed correlation may be due to the fact that pulmonary function measures do not accurately represent degree of parenchymal involvement in many patients with sarcoidosis. One study did identify a negative correlation between total lung capacity (TLC) and AHI, although TLC values in moderate-to-severe OSA remained above 80% of predicted, suggesting minimal spirometric restriction [13].

The role of corticosteroid therapy in the pathogenesis of OSA among people with sarcoidosis remains inconclusive. Two studies reported a higher prevalence of corticosteroid use in patients with concomitant OSA, potentially implicating steroid-induced weight gain and fat redistribution in upper airway collapsibility [11, 13]. In contrast, two other studies found no significant association between corticosteroid therapy and OSA risk, highlighting the need for more rigorous, controlled investigations into this potential mechanism [12, 16].

### Strengths and limitations:

One of the strengths of this review is its inclusion of recent cross-sectional studies, offering a more robust estimate of OSA prevalence in sarcoidosis compared to earlier literature [39]. Unlike that review, we excluded studies in which data were collected retrospectively and conducted a stratified analysis of OSA risk based on both conventional and sarcoidosis-specific factors, an approach, to our knowledge, not undertaken in previous reviews. Another strength is that we only included studies where both sarcoidosis and OSA status were fully ascertained, addressing concerns that OSA is often under-recognised in usual care and therefore unreliably captured in retrospective datasets. Furthermore, by including only studies with sarcoidosis diagnosed clinically or according to the international guidelines, we reduced misclassification bias, improving diagnostic specificity and ensuring that observed associations with OSA are based on accurately defined sarcoidosis populations. Together, these methodological strengths enhance the validity of our findings and provide a clearer understanding of the burden of OSA in people with sarcoidosis.

Nonetheless, several limitations should be acknowledged. Although this review was not conducted as a systematic review, a structured search and additional quality assessment were applied to ensure the robustness of included studies. Sample sizes in many studies were small and clinically heterogeneous, limiting the generalisability of findings. All studies recruited from either tertiary or university centres, and participation rates were generally unreported, further raising concerns about selection bias and external validity. Two studies did not perform laryngeal biopsies, which could have excluded potential SURT involvement, a potential contributor to OSA [15, 18]. Additionally, only one study excluded participants with neurosarcoidosis, a condition known to affect central sleep regulation via the hypothalamic sleep and wakefulness centre [16]. Further limitations include the use of HSAT in two studies [12, 13], which may underestimate OSA severity compared to gold-standard PSG and few studies distinguished between REM-predominant OSA and positional OSA, which may have different implications for treatment and pathophysiology. The reporting of corticosteroid use was inconsistent, and most studies did not include data on dosage or treatment duration, limiting interpretation of its role as a potential OSA risk factor.

Another limitation is the absence of data on inflammatory biomarkers, which may offer mechanistic insights into the link between sarcoidosis and OSA. A Swedish nested case-control study demonstrated that elevated inflammatory markers can precede the diagnosis of sarcoidosis [40]. IL-6, in particular, promotes Th1/Th17 differentiation and granuloma formation and is known to increase in response to poor sleep quality and fragmented sleep in otherwise healthy people [41, 42]. Furthermore, IL-6 and CDCP1 levels correlate with OSA severity, and CPAP therapy has not consistently suppressed IL-6 elevations in affected patients [43, 44]. Given these findings, it is biologically plausible that OSA may exacerbate immune activation in predisposed people, potentially amplifying the immunopathogenesis of sarcoidosis. Supporting this hypothesis, OSA has been associated with an increased risk of autoimmune conditions, including rheumatoid arthritis, systemic lupus erythematosus, and ankylosing spondylitis [45]. These associations raise the possibility that OSA may not only coexist with sarcoidosis but potentially influence its onset or clinical course.

### Future directions

Although current evidence suggests that OSA may be more prevalent in people with sarcoidosis, current studies are limited by small sample sizes and methodological heterogeneity. To better understand the interplay between sarcoidosis and OSA, future research should prioritise large, multicentre prospective studies using standardised diagnostic criteria for both conditions. Notably, while increased BMI in people with sarcoidosis was consistently associated with elevated OSA risk, the association with OSA risk and corticosteroid use was mixed. This raises an important clinical question: could the anti-inflammatory effects of corticosteroids, by reducing active sarcoidosis-related inflammation, offset the increased OSA risk associated with corticosteroid-induced weight gain?

An additional, underexplored hypothesis is that OSA itself may contribute to immune activation and accelerate sarcoidosis progression. However, whether comorbid OSA worsens clinical outcomes in sarcoidosis remains uncertain, as none of the included studies evaluated prognostic endpoints. Furthermore, while fibrotic ILDs such as idiopathic pulmonary fibrosis have been associated with a high nocturnal hypoxic burden and worse prognosis when comorbid with OSA [46], it is unclear whether sarcoidosis with OSA carries a similar risk. Hypoxic burden has emerged as a potentially superior biomarker of cardiovascular risk compared to AHI, but was not reported in any of the included studies [47]. Future prospective studies with larger, well-defined cohorts are essential to address these questions. These studies should control for confounders such as corticosteroid exposure, BMI, age, and radiological stage to accurately delineate risk factors and inform clinical management.

## Conclusion

The underlying pathophysiological link between sarcoidosis and OSA remains incompletely understood. This review suggests that the prevalence of OSA in sarcoidosis is considerably higher than previously estimated and shares the conventional risk factors of advancing age and increased BMI with the broader population. Given the overlapping features of these two conditions, clinicians should consider comorbid OSA in people sarcoidosis who present with unexplained fatigue or pulmonary hypertension.

## Data Availability

My manuscript has no associated data.
